# Follow-up care after treatment for prostate cancer: evaluation of a supported self-management and remote surveillance programme

**DOI:** 10.1186/s12885-019-5561-0

**Published:** 2019-04-23

**Authors:** Jane Frankland, Hazel Brodie, Deborah Cooke, Claire Foster, Rebecca Foster, Heather Gage, Jake Jordan, Ines Mesa-Eguiagaray, Ruth Pickering, Alison Richardson

**Affiliations:** 1 University of Southampton, School of Health Sciences, Highfield, Southampton, SO17 1BJ UK; 2 University of Surrey, School of Health Sciences, Guildford, Surrey, GU2 7XH UK; 3 Department of Clinical and Experimental Medicine, University of Surrey, Surrey Health Economics Centre, Guildford, Surrey, GU2 7XH UK; 4 University of Edinburgh, Usher Institute of Population Health Sciences and Informatics, Nine Edinburgh BioQuarter, Teviot Place, Edinburgh, EH8 9AG UK; 5 University of Southampton, Faculty of Medicine, Highfield, Southampton, SO17 1BJ UK; 6 University of Southampton, School of Health Sciences and University Hospital Southampton NHS Foundation Trust, Highfield, Southampton, SO17 1BJ UK

**Keywords:** Evaluation, Cancer, Follow-up care, Prostate, Patient-led, Patient-triggered, Supported self-management, Remote surveillance, Effectiveness, Cost-effectiveness

## Abstract

**Background:**

Alternative models of cancer follow-up care are needed to ameliorate pressure on services and better meet survivors’ long-term needs. This paper reports an evaluation of a service improvement initiative for the follow-up care of prostate cancer patients based on remote monitoring and supported self-management.

**Methods:**

This multi-centred, historically controlled study compared patient reported outcomes of men experiencing the new Programme with men experiencing a traditional clinic appointment model of follow-up care, who were recruited in the period immediately prior to the introduction of the Programme. Data were collected by self-completed questionnaires, with follow up measurement at four and eight months post-baseline. The primary outcome was men’s unmet survivorship needs, measured by the Cancer Survivors’ Unmet Needs Survey. Secondary outcomes included cancer specific quality of life, psychological wellbeing and satisfaction with care. The analysis was intention to treat. Regression analyses were conducted for outcomes at each time point separately, controlling for pre-defined clinical and demographic variables. All outcome analyses are presented in the paper. Costs were compared between the two groups.

**Results:**

Six hundred and twenty-seven men (61%) were consented to take part in the study (293 in the Programme and 334 in the comparator group.) Regarding the primary measure of unmet survivorship needs, 25 of 26 comparisons favoured the Programme, of which 4 were statistically significant. For the secondary measures of activation for self-management, quality of life, psychological well-being and lifestyle, 20 of 32 comparisons favoured the Programme and 3 were statistically significant. There were 22 items on the satisfaction with care questionnaire and 13 were statistically significant. Per participant costs (British pounds, 2015) in the 8 month follow up period were slightly lower in the programme than in the comparator group (£289 versus £327). The Programme was acceptable to patients.

**Conclusion:**

The Programme is shown to be broadly comparable to traditional follow-up care in all respects, adding to evidence of the viability of such models.

**Electronic supplementary material:**

The online version of this article (10.1186/s12885-019-5561-0) contains supplementary material, which is available to authorized users.

## Background

While there is a trend in developed countries of lengthening survival following cancer treatment [1, 2], cancer survivors are often left with symptoms, side effects and psychological concerns as a consequence of their treatment [3, 4]. For men who have been treated for prostate cancer this includes physical needs, such as urinary and bowel problems and hot flushes, and psychological needs related to sexual dysfunction [5–7]. The common approach to cancer follow-up care, of routine clinic/office-based appointments for all patients at pre-specified intervals, does not always lead to these needs being addressed [8, 9]. In addition, the increasing numbers of cancer survivors in follow-up care over a long period of time means a system of review involving direct contact for all patients is unsustainable in resource constrained health care systems such as the British National Health Service (NHS).

There have been efforts to find more efficient ways to deliver follow-up care, with testing of alternatives such as nurse led care, general practitioner led care, shared care and patient led care [10]. In England, current policy advocates a risk stratified approach for post-treatment cancer care, with remote surveillance and supported self-management for those who are at low risk of disease recurrence [11]. The recommended model for delivery of this self-management focussed pathway is a collection of interventions including holistic needs assessment, treatment summaries, rapid re-access to specialised care when indicated, and patient education and support [12]. There has been recognition of the relevance of such an approach to other international contexts [13].

To date, there is some evidence to show that remote surveillance together with patient initiated follow-up (variously referred to as patient triggered, patient-led, on demand, open access, spontaneous or point of need follow-up), is acceptable to patients [14–19], cost effective [17, 20], efficient [16], safe [15–17, 21, 22] and does not lead to an increase in use of GP services [18]. Only a small number of studies have considered the impact of such models on patient reported outcomes, showing no detrimental effects nor improvements in quality of life (QoL) [15, 17, 19] and no improvements in psychological wellbeing [15, 17, 23] .

This paper reports the evaluation of a service improvement initiative for men who have completed treatment for prostate cancer (the TrueNTH Supported Self-Management and Follow-up Care Programme, henceforth termed the Programme). The Programme is part of a global initiative to improve the survivorship outcomes of men with prostate cancer, funded by The Movember Foundation [24] and delivered in the United Kingdom (UK) in partnership with Prostate Cancer UK [25]. The Programme is focussed on providing post treatment follow-up care which is better tailored to men’s needs, which supports them to achieve their personal goals in relation to those needs and is cost-effective and scalable. This paper presents findings that relate to the impact of the Programme on patient reported outcomes including unmet need, symptom experience, quality of life, psychological wellbeing, activation to self-manage, fear of recurrence, healthy behaviours, patient satisfaction with care, as well as cost. The key questions addressed are: i) are patient reported outcomes better for men who are managed through the Programme compared to men in traditional appointment-based follow-up care; ii) are any improved outcomes maintained over time; and iii) what is the difference in cost between the Programme and traditional appointment-based follow-up care?

## Methods

Only brief descriptions of the Programme and the evaluation methods are provided here, further details can be accessed elsewhere [26].

### Setting

The Programme was evaluated in four prostate cancer treatment centres in the NHS in England. Three of the centres were involved in an expression of interest process and were selected according to readiness for implementation and to represent providers of urology services in both rural and urban areas. The fourth site had been involved in development and piloting of the Programme and was included in the evaluation to boost recruitment.

### Study design

The evaluation was historically controlled, comparing outcomes for men on the Programme with a comparator group of men recruited whilst attending a follow-up care appointment in the period prior to the introduction of the Programme. Outcome was assessed at four and eight months post-baseline. An embedded, within trial cost comparison analysis compared costs between the two groups from a health care provider perspective. A concurrent qualitative evaluation of factors that facilitated or inhibited implementation of the Programme will be reported separately.

### Eligibility

Men were eligible to take part in the evaluation if: their mode of primary treatment was radical prostatectomy (RP), radiotherapy (RT) or primary androgen deprivation therapy (PADT); they were assessed as suitable for the Programme by their clinical team, using predefined clinical eligibility criteria (see Additional File 1); they were within three years of completion of RP or RT, or within three years of commencement of PADT. Men were assessed for the programme from six weeks post RP or RT, or three months post commencement of PADT, and were reassessed periodically if they did not meet the criteria at their first assessment. Men with metastatic disease who met the clinical eligibility criteria were included in the Programme.

### Comparator

Men in the comparator group were managed according to the follow up care protocol in place at their treatment centre prior to the introduction of the Programme, and this varied by centre and by treatment group. National guidance informing this did not prescribe intervals of follow up, mode of follow up or professional involved. Consequently, each centre worked to a different frequency of follow up appointments (commonly for RP patients, 3 appointments during the first year, then 6 monthly in year 2 and then annually; RT patients commonly, 3 appointments during the first year, then annually; and for HT patients commonly between 3 and 6 monthly appointments), setting of the appointment (often face-to-face but some telephone appointments for some patients; all were followed up in secondary care), professional involved (most often a mix of nurse and consultant care), and overall duration of follow-up (varying by clinician and centre, between 5 and 15 years).

### The Programme

The Programme is designed to deliver personalised survivorship care through remote monitoring and supported self-management. The Programme is managed on a day-to-day basis by a healthcare worker, known as a support worker, who acts as the co-ordinator of the patient’s follow-up care and as first point of contact for any problems or queries. A uro-oncology Clinical Nurse Specialist (CNS) oversees this work, with overall patient responsibility remaining with the supervising urologist/oncologist. Men’s suitability for the Programme is determined according to agreed clinical criteria (Additional file 1). Men on the Programme do not have scheduled urology/oncology follow-up appointments; their follow-up care is instead facilitated through a bespoke Patient Online System (called My Medical Record), with access for both men and the prostate cancer team. Recurrence of prostate cancer is tracked through periodic blood samples to detect Prostate Specific Antigen (PSA). For men in the Programme, blood samples are taken at the man’s General Practice or at their hospital phlebotomy service and are transferred to the Online System directly from the pathology laboratory. Results are accessible to the patient through the Online System as soon as they are available, and they can also view other personal information such as treatment summaries and care plans, complete a Holistic Needs Assessment known as a Health MOT, or securely message their clinical team. PSA results and completed Health MOTs are reviewed by the team during ‘virtual clinics’, and contact made with the man if there is any concern. There is a system of rapid re-access to clinic if needed. Systems are in place for men who do not wish to use Information Technology. Men attend a four-hour face-to-face group self-management workshop to prepare them for the Programme. The workshop is run by the CNS and support worker, and aims to educate men about their follow-up care, consequences of treatment, important signs and symptoms of possible recurrence, healthy lifestyles and setting of health and wellbeing goals.

### Study integrity

Ethical approval was granted by the National Research Ethics Service, East of England – Cambridge South (reference number 11/EE/1021). Written consent was sought for participation in the study, with separate consent for access to medical records. As an evaluation of a service improvement initiative, trial registration was not required. Reporting of the study follows appropriate guidance [27, 28].

### Data collection

Patient reported outcome data were collected by postal questionnaire. Outcomes were measured using validated instruments where available, details of these are given below. Demographic characteristics were also collected. Medical details, including cancer stage, grade, date of diagnosis and treatment received, were collected from hospital records.

Resources related to the provision of the Programme and care of the comparator group were collected from the cancer centre teams, databases and finance managers. For the Programme, the clinical teams provided patient-level data regarding utilisation of follow-up services (virtual clinic appointments, telephone and electronic correspondence and face-to face appointments), workshop attendance, registration with the Patient Online System and details of other prostate cancer related hospital service use. Time related to Programme provision (such as delivery of the workshop, registration of men on the online portal and conduct of the health needs assessment) was reported by staff for the estimation of costs. Information on the follow-up care of the comparator group was extracted from treatment centre databases. Data on wider prostate cancer related health service use (primary care, secondary care, allied health professionals and community-based care) were collected from both groups via the questionnaire at four and eight months.

### Outcome measures

#### Cancer survivors’ unmet needs

The primary outcome was men’s unmet needs, as measured by a modified Cancer Survivors’ Unmet Needs Survey (CaSUN) [29]. The CaSUN [29] comprises 35 items within five domains: existential survivorship (that is, life perspective), comprehensive care, information, quality of life, and relationships, with an additional six items about positive life changes. This study has followed others in using a simplified four point response format [30] of no need, low need, moderate need and high need. The CaSUN can be scored as totals of strength of unmet need (possible scores of 0 to 140) and number of unmet needs (possible scores of 0–35), or for each of the five domains separately. The range of attainable values for each domain subscale are provided within the table of results. Higher scores indicate more need.

#### Treatment side effects

The Expanded Prostate Cancer Index Composite Short Form [31] (EPIC-26) was used to measure prostate cancer treatment related symptom and bother. The EPIC-26 comprises five subscales of urinary incontinence, urinary irritative/obstructive, bowel, sexual, and hormonal symptoms and bother. Each subscale is scored from 0 to 100, with higher scores indicating better function.

#### Cancer specific quality of life

Cancer specific quality of life was measured using the Functional Assessment of Cancer Therapy Scale (FACT-G) [32], which comprises physical, emotional, social and functional subscales, to calculate subscale scores and a total score (0 to 108). Higher scores indicate better functioning. Possible subscale scores are provided within the table of results.

#### Psychological wellbeing

The General Health Questionnaire (GHQ-12) [33] measures current mental health. The measure has two scoring methods: the 0111 giving a maximum total score of 12 and the 0123 method a maximum total score of 36. A higher score indicates worse mental health.

#### Fear of cancer recurrence

Fear of cancer recurrence was measured using the 2 item Worry of Cancer Scale [34], which is scored from 0 to 20 with a higher score indicating greater worry.

#### Activation for self-management

Activation to self-manage was measured with the short form Patient Activation Measure (PAM)® [35, 36]. This measure comprises 13 items and results in a total score with a maximum of 100, a higher score indicating greater activation.

#### General health behaviours

The Fruit and Vegetable Screening Tool [37] was used to measure healthy eating behaviours. This measure asks about fruit and vegetable consumption within a typical day and was scored from 0 to 10, with higher scores being better.

The Godin Leisure Time questionnaire [38] asks about leisure time physical activity as mild, moderate and strenuous activity, plus activity to work up a sweat, as number of units of 15 plus minutes per week. A total score is calculated (0 to 400 with higher being better), which can be used to classify respondents as active, moderately active and insufficiently active.

#### Service use

Fifteen questions about contact with health and community services for prostate cancer related issues and patient costs related to prostate cancer were developed for a previous evaluation in which two of the current authors were involved [39].

#### Satisfaction with care

Eleven questions regarding experience and acceptability of follow-up care were also developed for the previous evaluation [39].

### Analysis

#### Programme effectiveness

Descriptive statistics were used to compare baseline clinical and demographic characteristics of the programme and comparator groups, as well as to compare those completing both 4 and eight-month follow-up questionnaires with those lost to attrition. Outcome measure total scales and subscales were computed according to the guidelines for each instrument. When guidelines were not available, a prorated score was calculated when at least 75% of the items for a scale/subscale were present, otherwise the score was set as missing. Means and standard deviations were calculated for all available cases for each outcome measure at each time point, along with the change from baseline. Patient satisfaction data were analysed using Mann-Whitney U tests.

Regression analyses were conducted for outcome measures at both follow-up times separately, controlling for pre-specified variables: cancer centre, each outcome at baseline, age, type of treatment, educational attainment, time since diagnosis, living status, co-morbidity, employment status, and ethnicity.

Outcome comparisons were also obtained from a mixed model including an interaction between group and time so that separate programme comparisons were estimated at four and eight months. Results supported the initial regression analyses, with additional significant differences in seven of the outcomes at the eight month time point (not shown).

Three pre-specified sets of subgroup analysis were also conducted: age with pre-specified dichotomisation at 70 years; none versus one or more comorbidity; and being in the 20% most deprived areas according to the English Index of Multiple Deprivation [40]. Separate regression analysis for the difference in outcome between the programme and comparator groups at each time point, with the same controlling variables as used in the previous analyses, was repeated for each subgroup, and the interaction term between each specified subgroup and the programme versus comparator group factor was examined. Estimates of the difference between programme and comparator for each subgroup were examined when the interaction term was found to be significant (at the 5% level). Only one test of interaction was statistically significant (not shown).

For all analyses, terms were deemed statistically significant at the 5% level.

#### Economic evaluation

Costs of all items of service use for the programme and the comparator groups were calculated for each patient individually, based on the frequency of service use (Additional file 2) multiplied by the corresponding unit costs sourced from national tariffs [41] or finance managers (Additional file 3). Software and system costs were averaged and applied top down. Mean costs for each item and overall costs were compared between groups. Costs are reported in British pounds, 2015. The analyses were conducted on complete case data, that is respondents who returned service use data at all time points. In cases where some items of service use data were completed but others were missing, simple mean imputation stratified by group and cancer centre was used for the missing items. Health economics analyses were conducted using STATA14 [42] and Microsoft Excel 2013.

## Results

### Participants

**Fig. 1:**
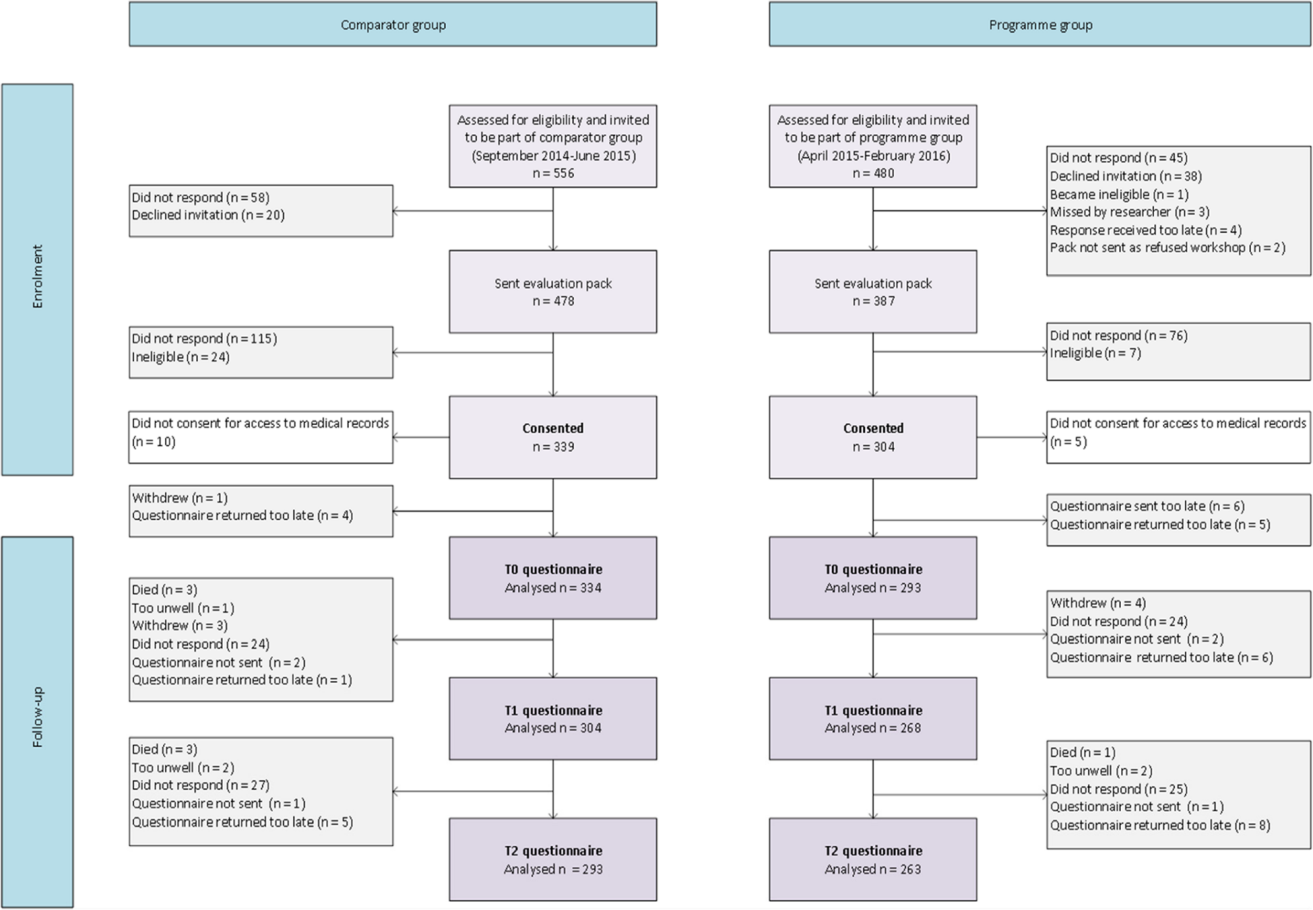
Flow diagram of study participation

The flow of participants through the study is shown in Fig. 1. Of the 1036 patients identified as eligible for the study, 627 (61%) completed a valid baseline questionnaire, and 522 (83%) of these also returned both follow-up questionnaires. Eight participants withdrew from the study and 12 died or became too unwell to continue. Fifteen men did not consent to use of medical records.

### Baseline characteristics

The programme and comparator groups were broadly similar on a range of socio-demographic and medical characteristics at baseline (Table 1). No characteristics were significantly different between the two groups, but there were slight differences in percentages of men in each group at three of the cancer centres. There were no significant differences in sociodemographic or medical characteristics between those who completed all three questionnaires and those lost to attrition (Additional file 4).

**Table 1:** Baseline characteristics of patients included in the study

	All	Programme group	Comparator group
	(*n* = 627)	(*n* = 293)	(*n* = 334)
Centre			
1	155 (25)	99 (34)	56 (17)
2	202 (32)	100 (34)	102 (31)
3	146 (23)	53 (18)	93 (28)
4	124 (20)	41 (14)	83 (25)
Ethnicity^a^			
White	607 (97)	281 (97)	326 (99)
Mixed	2 (0)	1 (0)	1 (0)
Asian	4 (1)	3 (1)	1 (0)
Black	4 (1)	4 (1)	0
Other	4 (1)	2 (1)	2 (1)
Missing	6 (1)	2 (1)	4 (1)
Qualifications^a^			
No qualifications	166 (27)	70 (24)	96 (30)
GCSE/O level	106 (17)	46 (16)	60 (18)
Vocational	131 (21)	63 (22)	68 (21)
A level	45 (7)	28 (10)	17 (5)
Undergraduate	54 (9)	27 (9)	27 (8)
Postgraduate	34 (6)	21 (7)	13 (4)
Other	81 (13)	35 (12)	46 (14)
Missing	10 (2)	3 (1)	7 (2)
Employment status^a^			
Retired	479 (77)	224 (77)	255 (77)
Employed full time	56 (9)	27 (9)	29 (9)
Employed part time	34 (5)	18 (6)	16 (5)
Employed on sick leave	5 (1)	1 (0)	4 (1)
Self employed	33 (5)	16 (6)	17 (5)
Disabled or long term sick	13 (2)	3 (1)	10 (3)
Unemployed	3 (0)	2 (1)	1 (0)
Missing	4 (1)	2 (1)	2 (1)
Marital status^a^			
Married/civil partnership	507 (81)	233 (80)	274 (82)
Widowed	36 (6)	19 (6)	17 (5)
Living with partner	33 (5)	17 (6)	16 (5)
Divorced/separated	31 (5)	15 (5)	16 (5)
Single	19 (3)	9 (3)	10 (3)
Missing	1 (0)	0	1 (0)
Living			
In own home	554 (88)	260 (89)	294 (88)
In rented home	57 (9)	29 (10)	28 (8)
Temporary accommodation	3 (1)	1 (0)	2 (1)
Other	13 (2)	3 (1)	10 (3)
Caring responsibilities for children or adults^a^			
Yes	75 (12)	41 (14)	34 (10)
No	548 (88)	251 (86)	297 (90)
Missing	4 (1)	1 (0)	3 (1)
Access to the internet at home^a^			
Yes	530 (85)	254 (87)	276 (83)
No	96 (15)	39 (13)	57 (17)
Missing	1 (0)	0	1 (0)
Index of Multiple Deprivation decile^a^			
1	25 (4)	13 (5)	12 (4)
2	28 (5)	15 (5)	13 (4)
3	51 (8)	21 (7)	30 (9)
4	88 (14)	39 (14)	49 (15)
5	89 (15)	36 (13)	53 (16)
6	75 (12)	34 (12)	41 (13)
7	64 (10)	34 (12)	30 (9)
8	72 (12)	41 (14)	31 (9)
9	70 (11)	29 (10)	41 (13)
10	53 (9)	24 (8)	29 (9)
Missing	12 (2)	7 (2)	5 (2)
Age of participant (in years)			
Mean (SD)	70 (7)	70 (7)	71 (7)
Min to max	44 to 91	45 to 84	44 to 91
Time since diagnosis (in years)^a^			
Mean (SD)	2 (2)	2 (2)	2 (2)
Min to max	0 to 14	0 to 14	0 to 14
*n*	623	292	331
Time from treatment^a^			
0–1 years	314 (51)	160 (56)	154 (47)
> 1–2 years	185 (30)	69 (24)	116 (35)
> 2–3 years	114 (19)	56 (20)	58 (18)
Missing	14 (2)	8 (3)	6 (2)
Number of comorbidities			
Mean (SD)	2 (1)	2 (1)	2 (1)
Min to max	0 to 6	0 to 5	0 to 6
Treatment^a^			
Radical prostatectomy	178 (29)	83 (29)	95 (29)
External Beam Radiotherapy (EBRT)	54 (9)	23 (8)	31 (9)
Hormone therapy (HT)	91 (15)	27 (9)	64 (19)
Brachytherapy (BT)	4 (1)	2 (1)	2 (1)
EBRT and HT	264 (43)	143 (50)	121 (37)
BT and HT	14 (2)	9 (3)	5 (2)
BT and EBRT	3 (1)	1 (0)	2 (1)
BT and EBRT and HT	12 (2)	1 (0)	11 (3)
Missing	7 (1)	4 (1)	3 (1)
T Stage^a^			
T1	64 (11)	32 (11)	32 (11)
T2	255 (44)	119 (42)	136 (45)
T3	237 (40)	118 (42)	119 (39)
T4	24 (4)	12 (4)	12 (4)
T stage unknown	4 (1)	1 (0)	3 (1)
Missing	43 (7)	11 (4)	32 (10)
N Stage^a^			
N0	528 (91)	262 (91)	266 (92)
N1	33 (6)	14 (5)	19 (7)
N stage unknown	17 (3)	12 (4)	5 (2)
Missing	49 (8)	5 (2)	44 (13)
M Stage^a^			
M0	551 (95)	273 (95)	278 (95)
M1	19 (3)	7 (2)	12 (4)
M stage unknown	12 (2)	8 (3)	4 (1)
Missing	45 (7)	5 (2)	40 (12)
PSA at diagnosis^a^			
Less than 10	287 (48)	142 (49)	145 (46)
10 to 20	163 (27)	83 (29)	80 (26)
More than 20	154 (25)	65 (22)	89 (28)
Missing	23 (4)	3 (1)	20 (6)
Risk stratification (3)			
Advanced (metastatic)	17 (3)	7 (2)	10 (3)
Localised high risk	65 (10)	31 (11)	34 (10)
Localised intermediate risk	183 (29)	96 (33)	87 (26)
Localised low risk	36 (6)	15 (5)	21 (6)
Localised risk unknown	8 (1)	3 (1)	5 (2)
Locally advanced	245 (39)	123 (42)	122 (37)
Insufficient data	73 (12)	18 (6)	55 (17)

^a^Percentages for non-missing categories calculated amongst cases with valid responses

### Study outcomes

#### Primary outcome –Cancer survivors’ unmet needs (CaSUN)

Results of the regression analyses for the CaSUN at each time point are shown in Table 2, detailing both scoring methods [29]. There were statistically significant reductions at the four-month follow-up for the programme compared to the comparator group in strength of unmet needs and total number of unmet needs (*p* = 0.025 and *p* = 0.020 respectively) and for the existential survivorship needs subscale (*p* = 0.041 for strength of needs and *p* = 0.022 for number of needs). These differences were reduced and not statistically significant at the eight-month follow-up point.

**Table 2:** Regression analysis of primary outcome measure (Cancer Survivors’ Unmet need – CaSUN [29] ) at 4 and 8 month follow up points by group

Outcome (Direction) Subscales (Range)					
Assessment	Programme group Mean (SD) *n* = 293	Comparator group Mean (SD) *n* = 334	Programme– comparator difference (95% CI)^a^	*P* value	Direction favours Programme
CaSUN Strength of need (higher = more need)					
Total score (0 to 140)					
Baseline	24.4 (17.2) *n* = 288	23.4 (17.7) *n* = 330			
4 months	18.3 (15.5) *n* = 262	20.6 (18.1) *n* = 296	−2.4 (−4.5, −0.3) *n* = 524	0.025	YES
8 months	17.9 (16.2) *n* = 260	19.7 (18.5) *n* = 286	−1.7 (−3.7, 0.3) *n* = 517	0.106	YES
Change (b-4 m)	4.8 *n* = 248	1.8 *n* = 292			
Change (b-8 m)	5.5 *n* = 251	3.4 *n* = 283			
Existential Survivorship (0 to 56)					
Baseline	4.8 (6.7) *n* = 290	4.5 (6.3) n = 330			
4 months	3.4 (5.6) *n* = 266	4.1 (6.0) *n* = 300	−0.7 (−1.4, −0.02) *n* = 533	0.041	YES
8 months	3.2 (5.6) *n* = 261	4.0 (6.3) *n* = 287	−0.6 (−1.3, 0.04) *n* = 519	0.065	YES
Change (b-4 m)	0.9 *n* = 254	0.1 n = 296			
Change (b-8 m)	1.1 *n* = 252	0.1 *n* = 284			
Comprehensive cancer care (0 to 24)					
Baseline	10.0 (5.5) *n* = 292	9.3 (5.9) *n* = 330			
4 months	8.0 (5.8) *n* = 262	8.2 (6.1) *n* = 299	−0.3 (−1.2, 0.4) *n* = 530	0.39	YES
8 months	7.7 (5.8) *n* = 260	7.8 (6.108) *n* = 287	−0.2 (−1.1, 0.5) *n* = 521	0.554	YES
Change (b-4 m)	1.9 *n* = 252	1.0 *n* = 295			
Change (b-8 m)	1.9 *n* = 253	1.5 *n* = 284			
Information (0 to 12)					
Baseline	3.4 (3.0) *n* = 291	3.5 (3.2) *n* = 330			
4 months	2.5 (2.9) *n* = 263	2.8 (3.0) n = 300	−0.1 (−0.6, 0.2) *n* = 531	0.462	YES
8 months	2.4 (2.8) *n* = 260	2.5 (2.8) *n* = 292	0.1 (−0.3, 0.5) n = 524	0.627	NO
Change (b-4 m)	0.8 *n* = 252	0.6 *n* = 296			
Change (b-8 m)	0.8 *n* = 252	0.9 *n* = 289			
Quality of life (0 to 8)					
Baseline	1.3 (1.5) *n* = 288	1.3 (1.6) *n* = 329			
4 months	0.9 (1.4) n = 266	1.1 (1.5) *n* = 300	−0.1 (−0.4, 0.06) *n* = 531	0.146	YES
8 months	0.9 (1.4) *n* = 259	1.1 (1.5) *n* = 287	−0.2 (− 0.4, 0.02) *n* = 517	0.083	YES
Change (b-4 m)	0.2 *n* = 253	0.08 *n* = 295			
Change (b-8 m)	0.3 *n* = 250	0.1 *n* = 284			
Relationships (0 to 12)					
Baseline	1.7 (2.1) *n* = 290	1.6 (2.0) *n* = 331			
4 months	1.3 (1.9) *n* = 264	1.5 (2.0) *n* = 298	−0.1 (− 0.4, 0.1) *n* = 531	0.283	YES
8 months	1.2 (1.9) n = 261	1.4 (1.9) *n* = 289	−0.1 (− 0.4, 0.08) *n* = 523	0.187	YES
Change (b-4 m)	0.2 *n* = 252	0.05 *n* = 295			
Change (b-8 m)	0.3 *n* = 253	0.1 *n* = 287			
CaSUN Unmet needs (higher = more need)					
Total number of unmet needs (0 to 35)					
Baseline	12.8 (8.2) *n* = 288	12.1 (8.6) *n* = 330			
4 months	10.1 (7.9) *n* = 262	11.1 (8.8) *n* = 296	−1.2 (−2.3, −0.2) *n* = 524	0.02	YES
8 months	10.0 (8.0) *n* = 260	10.9 (9.4) *n* = 286	−0.9 (−2.0, 0.1) *n* = 517	0.097	YES
Change (b-4 m)	2.0 *n* = 248	0.7 *n* = 292			
Change (b-8 m)	2.2 *n* = 251	1.1 *n* = 283			
Existential Survivorship (0 to 14)					
Baseline	3.3 (3.7) *n* = 290	3.1 (3.7) *n* = 330			
4 months	2.5 (3.4) *n* = 266	2.9 (3.6) n = 300	−0.524 (−0.9, −0.07) n = 533	0.022	YES
8 months	2.3 (3.2) *n* = 261	2.9 (3.9) n = 287	−0.4 (− 0.8, 0.05) n = 519	0.083	YES
Change (b-4 m)	0.5 n = 254	0.07 n = 296			
Change (b-8 m)	0.7 n = 252	0.08 n = 284			
Comprehensive Cancer Care (0 to 6)					
Baseline	4.2 (1.8) n = 292	3.8 (2.1) n = 330			
4 months	3.5 (2.1) n = 262	3.5 (2.2) n = 299	−0.1 (−0.4, 0.2) n = 530	0.437	YES
8 months	3.6 (2.2) n = 260	3.5 (2.3) n = 287	−0.07 (− 0.4, 0.2) n = 521	0.671	YES
Change (b-4 m)	0.5 n = 252	0.2 n = 295			
Change (b-8 m)	0.4 n = 253	0.3 n = 284			
Information (0 to 3)					
Baseline	1.7 (1.3) n = 291	1.6 (1.3) n = 330			
4 months	1.3 (1.3) *n* = 263	1.4 (1.3) *n* = 300	−0.03 (−0.2, 0.1) *n* = 531	0.78	YES
8 months	1.3 (1.3) *n* = 260	1.4 (1.3) *n* = 292	−0.01 (− 0.2, 0.2) *n* = 524	0.911	YES
Change (b-4 m)	0.3 *n* = 252	0.2 *n* = 296			
Change (b-8 m)	0.3 *n* = 252	0.2 *n* = 289			
Quality of life (0 to 2)					
Baseline	0.8 (0.8) *n* = 288	0.8 (0.8) *n* = 329			
4 months	0.6 (0.8) *n* = 266	0.7 (0.8) *n* = 300	−0.1 (−0.2, 0.02) *n* = 531	0.108	YES
8 months	0.6 (0.8) *n* = 259	0.7 (0.8) *n* = 287	−0.1 (− 0.2, 0.00) *n* = 517	0.064	YES
Change (b-4 m)	0.1 *n* = 253	0.05 *n* = 295			
Change (b-8 m)	0.1 *n* = 250	0.04 *n* = 284			
Relationships (0 to 3)					
Baseline	1.0 (1.0) *n* = 290	0.9 (1.0) *n* = 331			
4 months	0.7 (1.0) *n* = 264	0.8 (1.0) *n* = 298	−0.06 (−0.2, 0.08) *n* = 531	0.385	YES
8 months	0.7 (1.0) *n* = 261	0.8 (1.0) n = 289	−0.08 (− 0.2, 0.06) *n* = 523	0.263	YES
Change (b-4 m)	0.1 n = 252	0.07 *n* = 295			
Change (b-8 m)	0.1 n = 253	0.09 *n* = 287			
CaSUN Positive changes in life (higher = better)					
Number of positive changes (0 to 6)					
Baseline	2.0 (1.8) n = 288	1.9 (1.7) n = 331			
4 months	2.1 (1.9) *n* = 267	1.8 (1.8) *n* = 303	0.2 (−0.08, 0.5) *n* = 535	0.166	YES
8 months	2.1 (1.8) *n* = 259	1.6 (1.9) n = 288	0.221 (−0.1, 0.5) *n* = 517	0.179	YES
Change (b-4 m)	−0.06 *n* = 252	0.1 *n* = 300			
Change (b-8 m)	−0.04 *n* = 250	0.3 *n* = 284			

#### Secondary outcomes

Additional file 5 shows results for the secondary outcome measures. There were statistically significant improvements for the EPIC-26 bowel subscale for men in the programme group compared to the comparator group at both the four (*p* = 0.016) and eight-month (*p* = 0.003) follow-up points. A statistically significant improvement in mental health favouring the programme group was found for the GHQ-12 at four months using the most common 0111 scoring method [43] (*p* = 0.032) but not at eight months, and for neither time point using the alternative 0123 scoring method. There were no statistically significant differences between groups for any of the other secondary outcome scales/subscales at either time point.

### Patient satisfaction

Participant satisfaction data are presented in Table 3. The programme group reported significantly more satisfaction for nine of eleven statements at the four-month follow-up point. However, at eight months, only the statement ‘I have known who to contact with any problems’ showed a significant difference between the groups, with more programme group men strongly agreeing and more comparator group men agreeing to the statement.

**Table 3:** comparison of patient satisfaction with follow up care at 4 month and 8 month time points

	Strongly agree *N* (%)	Agree *N* (%)	Neither agree nor disagree *N* (%)	Disagree *N* (%)	Strongly disagree *N* (%)	Mann Whitney U	*p*-value
4 months							
I have felt reassured							
Programme group	73 (29)	124 (50)	51 (21)	0 (0)	0 (0)	27,356	0.000
Comparator group	56 (20)	119 (43)	81 (29)	8 (3)	12 (5)		
I have known who to contact with any problems							
Programme group	125 (50)	115 (46)	8 (3)	0 (0)	0 (0)	65,139	0.000
Comparator group	86 (31)	158 (57)	14 (5)	7 (2)	13 (5)		
I have felt comfortable about contacting the doctors and nurses with any problems							
Programme group	113 (46)	121 (48)	12 (5)	2 (1)	0 (0)	67,705	0.000
Comparator group	93 (33)	143 (51)	22 (8)	6 (2)	15 (6)		
I have felt isolated							
Programme group	1 (1)	6 (2)	18 (7)	85 (34)	138 (56)	37,907	0.019
Comparator group	7 (3)	11 (4)	34 (12)	91 (33)	133 (48)		
I have felt that the care received was thorough							
Programme group	103 (42)	116 (47)	27 (11)	0 (0)	1 (0)	67,212	0.000
Comparator group	80 (29)	134 (48)	48 (17)	6 (2)	12 (4)		
I have felt able to ask questions							
Programme group	112 (45)	127 (51)	9 (4)	1 (0)	0 (0)	67,944.50	0.000
Comparator group	93 (33)	143 (51)	26 (9)	4 (2)	14 (5)		
I have felt that the doctors/nurses spent enough time with me							
Programme group	97 (39)	117 (47)	31 (13)	1 (.5)	1 (.5)	68,095	0.000
Comparator group	78 (28)	137 (49)	41 (15)	7 (3)	17 (6)		
I have felt involved in decisions about my care							
Programme group	93 (38)	114 (46)	36 (15)	2 (1)	1 (0)	67,553.50	0.000
Comparator group	72 (26)	132 (47)	55 (19)	8 (3)	13 (5)		
Has the health care you received been acceptable	yes	no	unsure				
Programme group	230 (93)	0 (0)	18 (7)			35,521	0.278
Comparator group	251 (90)	4 (1)	24 (9)				
Has the health care you received met your expectations	exceeded	met	Fell short	unsure			
Programme group	36 (15)	186 (75)	8 (3)	17 (7)		36,827.50	0.033
Comparator group	25 (9)	213 (77)	13 (5)	25 (9)			
How would you rate the quality of care you have received	Excellent	Very good	Good	Fair	Poor		
Programme group	70 (29)	94 (38)	66 (27)	12 (5)	2 (1)	35,232	0.264
Comparator group	69 (25)	109 (40)	67 (25)	26 (9)	3 (1)		
8 months							
I have felt reassured							
Programme group	45 (19)	122 (50)	64 (26)	6 (3)	5 (2)	33,946	0.906
Comparator group	61 (23)	125 (45)	81 (29)	8 (2)	4 (1)		
I have known who to contact with any problems							
Programme group	98 (40)	139 (56)	6 (2)	1 (1)	3 (1)	30,686	0.015
Comparator group	85 (31)	173 (62)	11 (4)	6 (2)	3 (1)		
I have felt comfortable about contacting the doctors and nurses with any problems							
Programme group	91 (37)	132 (54)	17 (7)	1 (1)	3 (1)	31,896.50	0.207
Comparator group	86 (31)	168 (61)	15 (5)	5 (2)	3 (1)		
I have felt isolated							
Programme group	4 (2)	8 (3)	28 (11)	71 (29)	134 (55)	34,774	0.535
Comparator group	3 (1)	11 (4)	32 (12)	88 (32)	142 (51)		
I have felt that the care received was thorough							
Programme group	73 (30)	122 (51)	42 (17)	3 (1)	2 (1)	32,951	0.834
Comparator group	79 (29)	144 (52)	44 (16)	5 (2)	3 (1)		
I have felt able to ask questions							
Programme group	87 (36)	135 (55)	21 (9)	0 (0)	1 (0)	33,267.50	0.850
Comparator group	92 (34)	164 (60)	12 (4)	4 (1)	3 (1)		
I have felt that the doctors/nurses spent enough time with me							
Programme group	77 (32)	122 (50)	38 (16)	6 (2)	1 (0)	32,749	0.506
Comparator group	80 (29)	143 (52)	43 (15)	8 (3)	3 (1)		
I have felt involved in decisions about my care							
Programme group	74 (31)	120 (49)	45 (19)	3 (1)	1 (0)	32,089.50	0.317
Comparator group	72 (26)	146 (53)	50 (18)	7 (2)	2 (1)		
Has the health care you received been acceptable	yes	no	unsure				
Programme group	219 (91)	3 (1)	19 (8)			33,257.50	0.491
Comparator group	241 (89)	7 (3)	23 (9)				
Has the health care you received met your expectations	exceeded	met	Fell short	unsure			
Programme group	24 (10)	192 (80)	6 (2)	18 (8)		32,853	0.480
Comparator group	23 (9)	213 (80)	12 (5)	19 (7)			
How would you rate the quality of care you have received	Excellent	Very good	Good	Fair	Poor		
Programme group	51 (23)	85 (38)	74 (33)	10 (5)	3 (1)	28,942.50	0.913
Comparator group	63 (24)	101 (39)	71 (27)	23 (9)	3 (1)		

### Health economic analysis

Analyses were conducted on a complete case sample of 206 men in the programme group and 265 men in the comparator group. The direct costs of provision of follow-up care over the eight month period was £102 per patient for the Programme group (Table 4), compared to £59 per patient for the comparator group, with the higher cost being largely accounted for by the cost of the workshop (£63 per participant). Comparison of other prostate cancer related service use showed an appreciable difference between the two groups, with a mean cost (SD) of £186 (411) for the programme group and £268 (1020) for the comparator group. The difference was in part due to a small number of expensive inpatient events in the comparator group. Primary care use was similar for each group. A small number of men in the programme group attended urology service appointments to address problems that arose during the study period. Combining direct costs and costs of service use, the programme group had lower overall average costs of £289 per patient compared to £327 for the comparator group. The utilisation of service items that underlie these cost figures are shown in Additional file 2.

**Table 4:** Costs of service provision and service utilisation by group (£ stirling)

	Programme group (*N* = 206)				Comparator group (*N* = 265)			
	mean	sd	min	max	mean	sd	min	max
Total Cost								
	288.7	413.5	33.7	4185.0	327.3	1037.2	0	12,632.8
Subtotal								
Direct cost of Intervention	102.3	21.0	18.8	200.9	58.6	90.5	0	639.0
Total cost of Service use	186.3	411.4	0	4069.3	268.6	1020.7	0	12,499.4
Programme group follow up care costs								
Screening Cost	4.1	0.00	4.1	4.1				
Introduction to support worker	6.2	0.00	6.2	6.2				
Set up on PSA tracker system	5.0	0.00	5.0	5.0				
PSA Reviews	6.2	2.9	0	14.7				
Signed up to Online Portal	4.1	2.7	0	6.0				
Conducted Electronic Health Needs Assessment (HNA)	2.8	5.0	0	25.0				
Conducted Paper Health Needs Assessment (HNA)	2.4	1.5	0	3.4				
Staff Member electronic Messages	1.0	2.4	0	16.5				
Patient electronic Messages	0.8	2.4	0	21.0				
Cost of delivery of workshop (per patient)	62.9	11.8	0	65.1				
Comparator group follow up care costs								
Face to Face CLINIC, CNS Band6					1.5	5.8	0	42.0
Face to Face CLINIC, CNS Band7					3.6	9.0	0	52.0
Face to Face CLINIC, CNS Band8A					0.1	1.1	0	10.3
Face to Face CLINIC, Registrar Urology					7.4	18.7	0	125.3
Face to Face CLINIC, Consultant Urology					10.8	35.0	0	200.0
Face to Face CLINIC, Registrar Oncology					0.1	1.9	0	31.3
Face to Face CLINIC, Consultant Oncology					29.6	81.4	0	600.0
Telephone CLINIC, CNS Band6					0.00	0.00	0	0
Telephone CLINIC, CNS Band7					3.0	7.9	0	39.0
Telephone CLINIC, CNS Band8A					0.08	1.2	0	20.6
Telephone CLINIC, Consultant Oncology					0.6	7.3	0	100.0
Unplanned Clinical calls cost								
Telephone Contact, Support worker	3.4	7.2	0	36.5	0.04	0.4	0	5.0
Telephone Contact, CNS Band6	0.07	0.6	0	7.0	0.00	0	0	0
Telephone Contact, CNS Band7	0.9	3.6	0	26.0	1.4	5.8	0	60.6
Telephone Contact, CNS Band8A	0.00	0.00	0	0	0.1	1.1	0	15.5
Telephone Contact, Registrar Urology	1.0	6.4	0	62.6	0.00	0.00	0	0
Telephone Contact, Consultant Urology	0.1	2.3	0	33.3	0.00	0.00	0	0
Telephone Contact, Consultant Oncology	0.8	6.1	0	66.6	0.00	0.00	0	0
Other prostate related service use								
GP Visit	48.2	63.1	0	396.0	53.1	72.5	0	396.0
GP Tel Advice	1.0	3.4	0	18.4	1.2	3.2	0	23.0
GP Home Visit	1.7	12.7	0	92.0	1.3	11.2	0	92.0
GP Nurse Visit	16.7	18.1	0	111.4	15.5	18.3	0	89.1
GP Nurse Tel advice	1.4	4.4	0	31.6	1.1	3.8	0	23.7
GP Nurse home visit	1.8	22.1	0	315.8	1.2	8.9	0	98.7
Social worker visit	0.7	7.7	0	79.0	1.7	13.6	0	158.0
Physiotherapist visit NHS	2.8	16.7	0	192.0	5.6	42.1	0	512.0
Dietician visit NHS	1.4	7.9	0	64.0	1.8	21.9	0	352.0
Counsellor Visit NHS	0.6	5.0	0	42.0	3.4	43.2	0	672.0
Psychiatrist/psychologist Visit NHS	0.4	4.1	0	42.0	0.1	2.5	0	42.0
Complementary Therapist Visit NHS	1.1	8.4	0	80.0	0.1	1.3	0	16.0
Services Helpline call	0.1	1.0	0	10.5	0.4	2.7	0	31.5
Attended Urology Clinic	37.8	86.0	0	464.9	0.00	0.00	0	0
Other Clinic Cost	20.4	70.1	0	468.0	30.7	130.5	0	1560.0
A&E	9.5	137.6	0	1976.0	26.1	199.9	0	1976.0
Hospital Day Case	31.1	162.1	0	1426.0	64.5	340.8	0	2852.0
Hospital Inpatient Cost	8.9	95.7	0	1232.0	59.3	534.8	0	5800.0
Ambulance use	0.00	0.00	0	0	0.7	8.5	0	98.0

## Discussion

The increasing number of cancer survivors has resulted in a need to develop more sustainable models of cancer follow-up care which deal with capacity issues faced by healthcare services and also better address the range of cancer-related problems that survivors face. The Programme evaluated here integrates remote surveillance with supported self-management in a follow-up care pathway which aims to provide men with care that better targets their needs, as well as to reduce numbers of clinic appointments. This study is, to our knowledge, the first published evaluation of such a pathway for men with prostate cancer. The Programme differs from most other remote follow-up models in that it has a focus on supported self-management and potential patient entry as early as 6 weeks post-treatment.

The Programme showed some positive influence on men’s cancer related problems, with analyses showing significantly greater improvement in the programme group in unmet survivorship needs, bowel problems and psychological wellbeing at the four-month follow-up point, though only the improvement in bowel problems was still found at eight months. Overall, men in the programme group tended to rate their outcomes more highly than men in the comparator group.

Of those outcomes which showed a positive influence, only the EPIC-26 measure has published minimally important differences (MID) [44]. Differences related to the bowel function and bother subscale for the Programme are below the MID of 4–6, reaching 2.7 at four months and 3.6 at eight months.

One reason for the small number of outcomes showing a significant influence of the Programme may be the relatively low rates of need and high functioning and quality of life at baseline, possibly arising from the fact that men are only eligible for the Programme when the cancer care team is satisfied that such issues have been resolved as far as possible. The notable exceptions to this are EPIC-26 sexual and hormonal subscales, for which normative means of 61.4 and 91.7 [45] respectively compare to baseline means of 20.7 and 77.7 for the programme group and 18.4 and 78.1 for the comparator group. The Programme did not result in significant change in these subscales, which suggests that further intervention development is required for these issues.

In line with other studies [14–19], satisfaction with the Programme was high. This was most notable at the four-month follow-up point, shortly after men’s attendance at the Programme workshop. It is possible that the intensive time with the nursing team and with peers that the workshop affords men is the reason for this.

While the cost of delivering the Programme was slightly greater than care provided to the comparator group, reduced service use costs in the programme group offset this additional expense. The evaluation only measured outcomes over an eight-month period, whilst men will be followed up by their cancer service for at least five, and sometimes ten, years. It would not be appropriate to extrapolate the findings into the future, since there are no data available about men’s service use in the longer term. However, evaluation of costs over a longer time period would spread the upfront costs of the Programme.

This study suggests that a shift to remote surveillance coupled with support for self-management is broadly comparable to appointments-based follow-up care in terms of patient reported outcomes. This supports other evaluations of similar pathways with different cancers [14–19, 21], that cancer follow-up care can be provided remotely with no detriment to patient outcomes. In addition, such a model is acceptable to patients and is largely cost neutral.

There are a number of limitations to the evaluation that warrant discussion. First, being a service improvement initiative, it was not possible or desirable to conduct a randomised controlled trial, with the concomitant requirements for strict control by researchers over participant inclusion and exclusion and highly controlled programme delivery. Instead, a non-random comparator cohort was used and a pragmatic approach was taken [46], allowing for clinical judgement on a patient’s suitability for the Programme and for flexibility in how Programme delivery responded to local context. While the programme and comparator groups proved to be similar at baseline on all measured characteristics there remains a possibility, without randomisation, of relevant baseline differences between the groups on aspects that were not measured [47]. Second, in order to reflect the complex nature of the intervention and its theoretical underpinning, the evaluation made use of multiple outcome measures. The analyses presented in this paper involved a total of 26 tests of significance for the primary outcome and 54 for the secondary outcomes across the two follow-up time points. We did not make correction for multiple testing. Overall, however, 17 (21%) of 80 comparisons were found to be statistically significant, where this degree of multiple testing would be expected to yield only four statistically significant differences due to chance [48]. Third, as men in the comparator group were treated by the same healthcare team as the programme group, there is a possibility that they experienced some elements of the Programme, such as contact with the support worker or an enhanced personalised approach because of Programme training, during the follow-up period. However, the clinical teams were made aware of this possibility and asked to minimise this as far as possible. Also, the fact that the introduction of the Programme boosted clinic capacity may have improved the experience of those in the comparator group. Fourth, the evaluation did not assess the ability of the Programme to detect progression or recurrence. Fifth, while men with metastatic disease who met the clinical eligibility criteria were included in the Programme, 95% of the sample comprised men with non-metastatic disease. It is therefore uncertain whether the Programme can be recommended for men with metastatic prostate cancer. Sixth, while Programme cost and hospital service use data were collected directly from the clinical teams involved with the study, the health economics analysis relied on patient self-reported data regarding use of wider, community-based health care, so may have been affected by problems with recall. Finally, some activity related to remote surveillance does not have an NHS reference cost for use in health economic analyses, and a compromise was made to pro rata other costs to represent these activities.

## Conclusions

This evaluation adds to the evidence base around remote surveillance, or patient-led follow-up, of post-treatment cancer patients, showing that a shift to a supported self-management model of care with remote surveillance and a transition workshop is at least comparable to appointment based follow-up care in terms of patient reported outcomes, is acceptable to men and is largely cost neutral in the first eight months. The model addresses capacity issues by replacing a ‘one size fits all’ model of scheduled direct contact with remote surveillance for men who are progressing well, and places emphasis on the patient role in recovery and maintenance of health and wellbeing, which is absent in the more traditional model. Current policy for England [11] recommends such an approach for cancer patients at low risk of recurrence, and this evaluation shows the Programme to be a viable model. Future research to assess impact and cost-effectiveness over a longer follow-up period would be valuable.

## Additional files

Additional file 1: Programme’s clinical eligibility criteria. Table containing the clinical eligibility criteria for inclusion in the Programme. (DOCX 12 kb)

Additional file 2: Frequency of service use for programme and comparator groups. Table containing frequency of service use data used to calculate cost of service delivery. (DOCX 18 kb)

Additional file 3: Unit costs used in calculation of provision of follow-up care and other prostate cancer related service use. Table containing unit costs and source of unit costs used to calculate cost of service delivery. (DOCX 15 kb)

Additional file 4: Comparison of baseline characteristics for those completing all three questionnaires and those lost to attrition. Table containing data comparing those who completed all three questionnaires and those lost to attrition. (DOCX 18 kb)

Additional file 5: Regression analysis of secondary outcome measures at 4 and 8 month follow up points by group. Table containing data for the secondary outcomes measures (Patient Activation Measure®, EPIC-26, FACT-G, GHQ-12, Worry of Cancer scale, and lifestyle measurements). (DOCX 20 kb)

## Abbreviations

CaSUN: Cancer Survivors’ Unmet Needs Survey
CNS: Clinical Nurse Specialist
EPIC-26: Expanded Prostate Cancer Index Composite Short Form
FACT-G: Functional Assessment of Cancer Therapy Scale
GHQ-12: The General Health Questionnaire
NHS: National Health Service (NHS)
PADT: primary androgen deprivation therapy
PAM: Patient Activation Measure
PSA: Prostate Specific Antigen
QoL: quality of life
RP: radical prostatectomy
RT: radiotherapy
UK: United Kingdom
