# Supplementary material for: Follow-up care after treatment for prostate cancer: evaluation of a supported self-management and remote surveillance programme
Source: BMC Cancer. 2019 Apr 23;19:368. doi: 10.1186/s12885-019-5561-0 (PMC6480799; doi:10.1186/s12885-019-5561-0)
Supplement: Supplementary file 1 — Programme’s clinical eligibility criteria. Table containing the clinical eligibility criteria for inclusion in the Programme. (DOCX 12 kb) [file 12885_2019_5561_MOESM1_ESM.docx]

**Additional file 1: Programme’s clinical eligibility criteria**

| **Treatment** | **Time since treatment** | **PSA level** | **Clinician’s assessment of functioning** |
| --- | --- | --- | --- |
|  |  |  |  |
| Radical prostatectomy | more than 6 weeks post surgery | ≤ 0.1 | functionally and emotionally stable |
|  |  |  |  |
| Radiotherapy | more than 6 weeks post surgery | < 2 | functionally and emotionally stable |
|  |  |  |  |
| Primary Androgen Deprivation therapy | more than 3 post months commencement of treatment | < 4 | functionally and emotionally stable |
