# Supplementary material for: Follow-up care after treatment for prostate cancer: evaluation of a supported self-management and remote surveillance programme
Source: BMC Cancer. 2019 Apr 23;19:368. doi: 10.1186/s12885-019-5561-0 (PMC6480799; doi:10.1186/s12885-019-5561-0)
Supplement: Supplementary file 2 — Frequency of service use for programme and comparator groups. Table containing frequency of service use data used to calculate cost of service delivery. (DOCX 18 kb) [file 12885_2019_5561_MOESM2_ESM.docx]

**Additional file 2: Frequency of service use for programme and comparator groups**

|  | **Programme group** | | | | | **Comparator group** | | | | |
| --- | --- | --- | --- | --- | --- | --- | --- | --- | --- | --- |
|  | **N** | **mean** | **sd** | **min** | **max** | **N** | **mean** | **sd** | **min** | **max** |
| **Subtotals** |  |  |  |  |  |  |  |  |  |  |
| Unplanned Clinical calls Number | 206 | 0.8 | 1.5 | 0 | 11 | 265 | 0.1 | 0.4 | 0 | 6 |
| Unplanned Clinical calls Duration | 206 | 8.3 | 15.3 | 0 | 80 | 265 | 1.8 | 6.9 | 0 | 70 |
| Telephone clinics Number | 206 |  |  |  |  | 265 | 0.2 | 0.6 | 0 | 3 |
| Telephone clinics Duration | 206 |  |  |  |  | 265 | 3.6 | 9.2 | 0 | 45 |
| Face2Face_clinics Number | 206 |  |  |  |  | 265 | 0.9 | 1.1 | 0 | 9 |
| Face2Face_clinics Duration | 206 |  |  |  |  | 265 | 13.7 | 17.6 | 0 | 135 |
| **Programme group follow up care costs** |  |  |  |  |  |  |  |  |  |  |
| PSA Reviews Number | 206 | 1.7 | 0.8 | 0 | 4 | 265 |  |  |  |  |
| Signed up to Online Portal | 206 | 0.6 | 0.4 | 0 | 1 | 265 |  |  |  |  |
| Conducted Electronic Health Needs Assessment (HNA) Number | 206 | 0.4 | 0.8 | 0 | 4 | 265 |  |  |  |  |
| Conducted Paper Health Needs Assessment (HNA) Number | 206 | 0.3 | 0.2 | 0 | 1 | 265 |  |  |  |  |
| Staff Member electronic Messages Number | 206 | 0.6 | 1.6 | 0 | 11 | 265 |  |  |  |  |
| Patient electronic Messages Number | 206 | 0.5 | 1.6 | 0 | 14 | 265 |  |  |  |  |
| **Comparator group follow up care costs** |  |  |  |  |  |  |  |  |  |  |
| Face to Face CLINIC, CNS Band6 Number |  |  |  |  |  | 265 | 0.08 | 0.3 | 0 | 2 |
| Face to Face CLINIC, CNS Band6 Duration |  |  |  |  |  | 265 | 2.1 | 8.3 | 0 | 60 |
| Face to Face CLINIC, CNS Band7 Number |  |  |  |  |  | 265 | 0.2 | 0.5 | 0 | 3 |
| Face to Face CLINIC, CNS Band7 Duration |  |  |  |  |  | 265 | 4.1 | 10.4 | 0 | 60 |
| Face to Face CLINIC, CNS Band8A Number |  |  |  |  |  | 265 | 0.02 | 0.1 | 0 | 1 |
| Face to Face CLINIC, CNS Band8A Duration |  |  |  |  |  | 265 | 0.1 | 1.1 | 0 | 10 |
| Face to Face CLINIC, Registrar Urology Number |  |  |  |  |  | 265 | 0.2 | 0.5 | 0 | 2 |
| Face to Face CLINIC, Registrar Urology Duration |  |  |  |  |  | 265 | 1.1 | 2.9 | 0 | 20 |
| Face to Face CLINIC, Consultant Urology Number |  |  |  |  |  | 265 | 0.1 | 0.4 | 0 | 2 |
| Face to Face CLINIC, Consultant Urology Duration |  |  |  |  |  | 265 | 1.6 | 5.2 | 0 | 30 |
| Face to Face CLINIC, Registrar Oncology Number |  |  |  |  |  | 265 | 0.00 | 0.06 | 0 | 1 |
| Face to Face CLINIC, Registrar Oncology Duration |  |  |  |  |  | 265 | 0.02 | 0.3 | 0 | 5 |
| Face to Face CLINIC, Consultant Oncology Number |  |  |  |  |  | 265 | 0.3 | 0. | 0 | 6 |
| Face to Face CLINIC, Consultant Oncology Duration |  |  |  |  |  | 265 | 4.4 | 12.2 | 0 | 90 |
| Telephone CLINIC, CNS Band6 Number |  |  |  |  |  | 265 | 0 | 0 | 0 | 0 |
| Telephone CLINIC, CNS Band6 Duration |  |  |  |  |  | 265 | 0 | 0 | 0 | 0 |
| Telephone CLINIC, CNS Band7 Number |  |  |  |  |  | 265 | 0.2 | 0.6 | 0 | 3 |
| Telephone CLINIC, CNS Band7 Duration |  |  |  |  |  | 265 | 3.5 | 9.1 | 0 | 45 |
| Telephone CLINIC, CNS Band8A Number |  |  |  |  |  | 265 | 0.01 | 0.1 | 0 | 2 |
| Telephone CLINIC, CNS Band8A Duration |  |  |  |  |  | 265 | 0.08 | 1.2 | 0 | 20 |
| Telephone CLINIC, Consultant Oncology Number |  |  |  |  |  | 265 | 0.01 | 0.09 | 0 | 1 |
| Telephone CLINIC, Consultant Oncology Duration |  |  |  |  |  | 265 | 0.09 | 1.1 | 0 | 15 |
| **Unplanned Clinical calls cost** |  |  |  |  |  |  |  |  |  |  |
| Telephone Contact, Support worker Number | 206 | 0.6 | 1.4 | 0 | 10 | 265 | 0.01 | 0.09 | 0 | 1 |
| Telephone Contact, Support worker Duration (mins) | 206 | 6.8 | 14.5 | 0 | 73 | 265 | 0.08 | 0.8 | 0 | 10 |
| Telephone Contact, CNS Band6 Number | 206 | 0.01 | 0.1 | 0 | 1 | 265 | 0 | 0 | 0 | 0 |
| Telephone Contact, CNS Band6 Duration | 206 | 0.1 | 0.9 | 0 | 10 | 265 | 0 | 0 | 0 | 0 |
| Telephone Contact, CNS Band7 Number | 206 | 0.09 | 0.3 | 0 | 3 | 265 | 0.1 | 0.4 | 0 | 6 |
| Telephone Contact, CNS Band7 Duration | 206 | 1.0 | 4.1 | 0 | 30 | 265 | 1.6 | 6.7 | 0 | 70 |
| Telephone Contact, CNS Band8A Number | 206 | 0 | 0 | 0 | 0 | 265 | 0.01 | 0.1 | 0 | 1 |
| Telephone Contact, CNS Band8A Duration | 206 | 0 | 0 | 0 | 0 | 265 | 0.1 | 1.1 | 0 | 15 |
| Telephone Contact, Registrar Urology Number | 206 | 0.03 | 0.2 | 0 | 2 | 265 | 0 | 0 | 0 | 0 |
| Telephone Contact, Registrar Urology Duration | 206 | 0.1 | 1.0 | 0 | 10 | 265 | 0 | 0 | 0 | 0 |
| Telephone Contact, Consultant Urology Number | 206 | 0.0 | 0.0 | 0 | 1 | 265 | 0 | 0 | 0 | 0 |
| Telephone Contact, Consultant Urology Duration | 206 | 0.02 | 0.3 | 0 | 5 | 265 | 0 | 0 | 0 | 0 |
| Telephone Contact, Consultant Oncology Number | 206 | 0.02 | 0.1 | 0 | 1 | 265 | 0 | 0 | 0 | 0 |
| Telephone Contact, Consultant Oncology Duration | 206 | 0.1 | 0.9 | 0 | 10 | 265 | 0 | 0 | 0 | 0 |
| **Other prostate related service use** |  |  |  |  |  |  |  |  |  |  |
| GP Visit Number | 206 | 1.3 | 1.7 | 0 | 11 | 265 | 1.4 | 2.0 | 0 | 11 |
| GP Tel Advice Number | 206 | 0.2 | 0.7 | 0 | 4 | 265 | 0.2 | 0.7 | 0 | 5 |
| GP Home Visit Number | 206 | 0.02 | 0.1 | 0 | 1 | 265 | 0.02 | 0.1 | 0 | 1 |
| GP Nurse Visit Number | 206 | 1.5 | 1.6 | 0 | 10 | 265 | 1.4 | 1.6 | 0 | 8 |
| GP Nurse Tel advice Number | 206 | 0.1 | 0.5 | 0 | 4 | 265 | 0.1 | 0.4 | 0 | 3 |
| GP Nurse home visit Number | 206 | 0.09 | 1.1 | 0 | 16 | 265 | 0.06 | 0.4 | 0 | 5 |
| Social worker visit Number | 206 | 0.01 | 0.1 | 0 | 1 | 265 | 0.02 | 0.1 | 0 | 2 |
| Physiotherapist visit Number | 206 | 0.09 | 0.5 | 0 | 6 | 265 | 0.1 | 1.3 | 0 | 16 |
| Dietician visit Number | 206 | 0.04 | 0.2 | 0 | 2 | 265 | 0.06 | 0.6 | 0 | 11 |
| Counsellor Visit Number | 206 | 0.01 | 0.1 | 0 | 1 | 265 | 0.1 | 1.0 | 0 | 16 |
| Psychiatrist/psychologist Visit Number | 206 | 0.01 | 0.1 | 0 | 1 | 265 | 0.00 | 0.06 | 0 | 1 |
| Complementary Therapist Visit Number | 206 | 0.07 | 0.53 | 0 | 5 | 265 | 0.01 | 0.09 | 0 | 1 |
| Services Helpline call Number | 206 | 0.01 | 0.1 | 0 | 1 | 265 | 0.04 | 0.2 | 0 | 3 |
| Attended Urology Clinic Number | 206 | 0.4 | 0.9 | 0 | 5 | 265 | 0 | 0 | 0 | 0 |
| Other Clinic Attendance (Entry 1) Number | 206 | 0.1 | 0.4 | 0 | 3 | 265 | 0.2 | 0.8 | 0 | 10 |
| Other Clinic Attendance (Entry 2) Number | 206 | 0.01 | 0.1 | 0 | 1 | 265 | 0.01 | 0.1 | 0 | 2 |
| Attended A&E Number | 206 | 0.01 | 0.1 | 0 | 2 | 265 | 0.03 | 0.2 | 0 | 2 |
| Attended Hospital Day Case Number | 206 | 0.04 | 0.2 | 0 | 2 | 265 | 0.09 | 0.4 | 0 | 4 |
| Attended Hospital Inpatient Number | 206 | 0.01 | 0.1 | 0 | 2 | 265 | 0.03 | 0.2 | 0 | 2 |
| Attended Hospital Inpatient Number of days | 206 | 0.01 | 0.1 | 0 | 2 | 265 | 0.1 | 1.4 | 0 | 22 |
| Number of ambulance uses | 206 | 0 | 0 | 0 | 0 | 265 | 0.01 | 0.09 | 0 | 1 |
