# Supplementary material for: Follow-up care after treatment for prostate cancer: evaluation of a supported self-management and remote surveillance programme
Source: BMC Cancer. 2019 Apr 23;19:368. doi: 10.1186/s12885-019-5561-0 (PMC6480799; doi:10.1186/s12885-019-5561-0)
Supplement: Supplementary file 3 — Unit costs used in calculation of provision of follow-up care and other prostate cancer related service use. Table containing unit costs and source of unit costs used to calculate cost of service delivery. (DOCX 15 kb) [file 12885_2019_5561_MOESM3_ESM.docx]

| **Additional file 3: Unit costs used in calculation of provision of follow-up care and other prostate cancer related service use** | | | | |
| --- | --- | --- | --- | --- |
| **Item** | **Cost (£)** | **Unit** | **Source** | **Comment** |
| Band 4 community support worker | 30 | per hour | 9. Scientific and professional staff. PSSRU 2016 |  |
| Band 6 CNS | 42 | per hour | 9. Scientific and professional staff. PSSRU 2016 |  |
| Band 7 CNS | 52 | per hour | 9. Scientific and professional staff. PSSRU 2016 |  |
| Band 8a CNS | 62 | per hour | 9. Scientific and professional staff. PSSRU 2016 |  |
| Registrar | 94 | Per consult | NHS Reference costs 2016 | Non-Consultant Led, follow up, face to face non admitted. Assumption on average 15 minute consult to apportion pro-rata. |
| Consultant, Medical | 100 | Per consult | NHS Reference costs 2016 | Consultant Led, follow up, face to face non admitted. Assumption on average 15 minute consult to apportion pro-rata. |
| GP visit | 36 | per 9.22 minute consult | 10.3b. PSSRU 2016. |  |
| GP email or Phone | 4.6 | per 4 minute intervention | 10.4 telephone triage. PSSRU |  |
| GP home visit | 92.2 | per 11.44 minute consult + 12 minute travel time | 10.3b. PSSRU 2016. | Used average consult length of 11.44 minutes plus 12 minute travel time as assumed in PSSRU 2015. 10.8a. GP hourly cost at £236 from PSSRU 2016 |
| Nurse visit | 11.14 | per 15.5 minutes | 10.2 Nurse (GP practice). PSSRU 2016 | used the average consult length of 15.5 minutes in GP practice for 10.6 Nurse PSSRU 2015. PSSRU 2016 £43/h |
| Nurse email or Phone | 7.9 | per 6.56 minute intervention | 10.4 telephone triage. PSSRU 2016 |  |
| Nurse home visit | 19.74 | per 15.5 minutes+12 minutes travel time | 10.2 Nurse (GP practice). PSSRU 2016 | used the average consult length of 15.5 minutes in GP practice for 10.6 Nurse PSSRU 2015. PSSRU 2016 £43/h. PSSRU 2015 noted travel time for GP home visits as 12 minutes. Added this to estimate consult cost |
| Social Worker visit | 79 | per hour | 11.2 Social worker (adult services) PSSRU 2016 | Assume 1 hour visit. No information available on average duration of a consult |
| Physio | 32 | per hour | 9. Scientific and professional staff. PSSRU 2016 | Assume 1 hour visit. Band 5 |
| Dietician | 32 | per hour | 9. Scientific and professional staff. PSSRU 2016 | Assume 1 hour visit. Band 5 |
| Counsellor | 42 | per hour | 9. Scientific and professional staff. PSSRU 2016 | Assume 1 hour visit. Band 6 |
| Psychiatrist | 42 | per hour | 9. Scientific and professional staff. PSSRU 2016 | Assume 1 hour visit. Band 6 |
| Complementary medicine | 16 |  |  | Average cost per month 8-28 pounds, 2013. "Prevalence of use of complementary and alternative medicine (CAM) by patients/consumers in the UK: systematic review of surveys" Clinical Medicine 2013, Vol 13, No 2: 126–31 |
| Helpline Service | 10.5 |  |  | Assume B6CNS 15 minute call |
| Cancer support group | 91.47 |  |  | //assume average outpatient urology clinic costs/ NHS Ref 2016, average of urology/oncology outpatient clinic cost |
| Urology/oncology clinic | 91.47 | per outpatient clinic | NHS REF costs 2016 |  |
| Urology/oncology advice PHONE | 10.5 |  |  | Assume B6CNS 15 minute call |
| Urology/oncology advice EMAIL | 10.5 |  |  | Assume B6CNS 15 activity |
| Hospital outpatient clinic, cancer | 156 | per outpatient visit | 7.1 NHS reference costs for hospital services. PSSRU 2016 | Overall average of all outpatient clinics. PSSRU 2016 |
| A&E | 988 | per visit | 8.2 PSSRU 2016 | Average cost for all users A&E and outpatient. 2009 uprated to 2016 for inflation |
| Hospital Day Case | 713 | per case | 7.1 NHS reference costs for hospital services. PSSRU 2016 |  |
| Hospital Inpatient Elective | 3653 | per case | 7.1 NHS reference costs for hospital services. PSSRU 2016 |  |
| Hospital Inpatient Non-elective short | 616 | per case | 7.1 NHS reference costs for hospital services. PSSRU 2016 |  |
| Hospital Inpatient Non-elective Long | 2900 | per case | 7.1 NHS reference costs for hospital services. PSSRU 2016 |  |
| Ambulance transit | 98 | per use | 7.1 NHS reference costs for hospital services. PSSRU 2016 | Overall average for all ambulance services (see and treat, refer, and convey. |
