# Supplementary material for: Follow-up care after treatment for prostate cancer: evaluation of a supported self-management and remote surveillance programme
Source: BMC Cancer. 2019 Apr 23;19:368. doi: 10.1186/s12885-019-5561-0 (PMC6480799; doi:10.1186/s12885-019-5561-0)
Supplement: Supplementary file 4 — Comparison of baseline characteristics for those completing all three questionnaires and those lost to attrition. Table containing data comparing those who completed all three questionnaires and those lost to attrition. (DOCX 18 kb) [file 12885_2019_5561_MOESM4_ESM.docx]

| **Additional file 4: Comparison of baseline characteristics for those completing all three questionnaires and those lost to attrition** | | |
| --- | --- | --- |
| **Patient characteristic** | **Participants with valid 4 and 8 months questionnaires N (%)** | **Participants lost to attrition N (%)** |
|  | **(n=522)** | **(n=105)** |
| **Group** |  |  |
| **Care Programme** | 236 (45) | 57 (54) |
| **Usual Care** | 286 (55) | 48 (46) |
| **Centre** |  |  |
| **1** | 137 (26) | 18 (17) |
| **2** | 168 (32) | 34 (32) |
| **3** | 122 (23) | 24 (23) |
| **4** | 95 (18) | 29 (28) |
| **Ethnicity*** |  |  |
| **White** | 506 (98) | 101 (97) |
| **Mixed** | 2 (0) | 0 |
| **Asian** | 3 (1) | 1 (1) |
| **Black** | 0 | 2 (2) |
| **Other** | 4 (1) | 0 |
| **Missing** | 5 (1) | 1 (1) |
| **Qualifications*** |  |  |
| **No qualifications** | 136 (26) | 30 (29) |
| **GCSE/O level** | 93 (18) | 13 (13) |
| **Vocational** | 106 (21) | 25 (25) |
| **A level** | 39 (8) | 6 (6) |
| **Undergraduate** | 46 (9) | 8 (8) |
| **Postgraduate** | 30 (6) | 4 (4) |
| **Other** | 65 (13) | 16 (16) |
| **Missing** | 7 (1) | 3 (3) |
| **Employment Status*** |  |  |
| **Retired** | 402 (78) | 77 (74) |
| **Employed full time** | 45 (9) | 11 (11) |
| **Employed part time** | 27 (5) | 7 (7) |
| **Employed on sick leave** | 3 (1) | 2 (2) |
| **Self employed** | 29 (6) | 4 (4) |
| **Disabled or long term sick** | 10 (2) | 3 (3) |
| **Unemployed** | 3 (1) | 0 |
| **Missing** | 3 (1) | 1 (1) |
| **Marital status*** |  |  |
| **Married/civil partnership** | 430 (83) | 77 (73) |
| **Widowed** | 27 (5) | 9 (9) |
| **Living with partner** | 27 (5) | 6 (6) |
| **Divorced/separated** | 23 (4) | 8 (8) |
| **Single** | 14 (3) | 5 (5) |
| **Missing** | 1 (0) | 0 |
| **Living** |  |  |
| **In own home** | 470 (90) | 84 (80) |
| **In rented home** | 40 (8) | 17 (16) |
| **Temporary accommodation** | 2 (0) | 1 (1) |
| **Other** | 10 (2) | 10 (3) |
| **Caring Responsibilities for children or Adults*** |  |  |
| **Yes** |  |  |
| **No** | 53 (10) | 22 (21) |
| **Missing** | 465 (90) | 83 (79) |
|  | 4 (1) | 0 |
| **Access to the internet at home*** |  |  |
| **Yes** | 442 (85) | 88 (84) |
| **No** | 79 (15) | 17 (16) |
| **Missing** | 1 (0) | 0 |
| **Index of Multiple Deprivation decile*** |  |  |
| **1** | 20 (4) | 5 (5) |
| **2** | 17 (3) | 11 (11) |
| **3** | 43 (8) | 8 (8) |
| **4** | 67 (13) | 21 (21) |
| **5** | 78 (15) | 11 (11) |
| **6** | 65 (13) | 10 (10) |
| **7** | 52 (10) | 12 (12) |
| **8** | 61 (12) | 11 (11) |
| **9** | 62 (12) | 8 (8) |
| **10** | 48 (9) | 5 (5) |
| **Missing** | 9 (2) | 3 (3) |
| **Age of participant (in years)** |  |  |
| **Mean (SD)** | 70 (7) | 70 (8) |
| **Min to max** | 45 to 85 | 45 to 91 |
| **Time since diagnosis (in years)*** |  |  |
| Mean (SD) | 2 (2) | 2 (2) |
| Min to max | 0 to 14 | 0 to 14 |
| n | 522 | 101 |
| **Number of comorbidities** |  |  |
| Mean (SD) | 2 (1) | 2 (1) |
| Min to max | 0 to 5 | 0 to 6 |
| **Time from treatment*** |  |  |
| 0-1 years | 267 (52) | 47 (48) |
| >1-2 years | 151 (29) | 34 (35) |
| >2-3 years | 97 (19) | 17 (17) |
| Missing | 7 (1) | 7 (7) |
| **Treatment*** |  |  |
| Radical prostactectomy | 156 (30) | 22 (21) |
| External Beam Radiotherapy (EBRT) | 42 (8) | 12 (12) |
| Hormone therapy | 72 (14) | 19 (18) |
| Brachytherapy (BT) | 3 (1) | 1 (1) |
| EBRT and hormone therapy | 221 (43) | 43 (42) |
| BT and hormone therapy | 11 (2) | 3 (3) |
| BT with EBRT | 2 (0) | 1 (1) |
| BT with EBRT and hormone therapy | 10 (2) | 2 (2) |
| Missing | 5 (1) | 2 (2) |
| **T Stage*** |  |  |
| T1 | 58 (12) | 6 (7) |
| T2 | 212 (43) | 43 (49) |
| T3 | 203 (41) | 34 (39) |
| T4 | 20 (4) | 4 (5) |
| TX | 4 (1) | 0 |
| Missing | 25 (5) | 18 (17) |
| **M Stage*** |  |  |
| M0 | 462 (94) | 89 (98) |
| M1 | 18 (4) | 1 (1) |
| MX | 11 (2) | 1 (1) |
| Missing | 31 (6) | 14 (13) |
| **N Stage*** |  |  |
| N0 | 450 (92) | 78 (89) |
| N1 | 26 (5) | 7 (8) |
| NX | 14 (3) | 3 (3) |
| Missing | 32 (6) | 17 (16) |
| **Risk stratification (3)** |  |  |
| Advanced (metastatic) | 17 (3) | 0 |
| Localised high risk | 59 (11) | 6 (6) |
| Localised intermediate risk | 152 (29) | 31 (30) |
| Localised low risk | 29 (6) | 7 (7) |
| Localised risk unknown | 6 (1) | 2 (2) |
| Locally advanced | 208 (40) | 37 (35) |
| Insufficient data | 51 (10) | 22 (21) |
| **PSA at diagnosis*** |  |  |
| Less than 10 | 240 (47) | 47 (49) |
| 10 to 20 | 139 (27) | 24 (25) |
| More than 20 | 129 (25) | 25 (26) |
| Missing | 14 (3) | 9 (9) |
| * Percentages for non-missing categories calculated amongst cases with valid responses | |  |
