# Supplementary material for: Follow-up care after treatment for prostate cancer: evaluation of a supported self-management and remote surveillance programme
Source: BMC Cancer. 2019 Apr 23;19:368. doi: 10.1186/s12885-019-5561-0 (PMC6480799; doi:10.1186/s12885-019-5561-0)
Supplement: Supplementary file 5 — Regression analysis of secondary outcome measures at 4 and 8 month follow up points by group. Table containing data for the secondary outcomes measures (Patient Activation Measure®, EPIC-26, FACT-G, GHQ-12, Worry of Cancer scale, and lifestyle measurements). (DOCX 20 kb) [file 12885_2019_5561_MOESM5_ESM.docx]

| **Additional file 5: Regression analysis of secondary outcome measures at 4 and 8 month follow up points by group** | | | | | |  |
| --- | --- | --- | --- | --- | --- | --- |
| **Outcome (reference) (direction)  Subscales (range)** | | | | | |  |
| **Assessment** | **Programme group** | **Comparator group** | **Programme group – comparator group difference (95% CI)*** | **P  value** | **Direction  favours Progra-- mme** | |
| **Patient Activation Measure ® (36, 37) (higher=better) (0 to 100)** | | | | | |  |
| Baseline | 63.02 (14.4) n=286 | 61.3 (14.1) n=323 |  |  |  | |
| 4 months | 64.3 (14.7) n=260 | 62.2 (14.6) n=289 | 1.2 (-0.8, 3.4)  n=514 | 0.246 | YES | |
| 8 months | 64.8 (15.6) n=255 | 63.5 (15.1) n=280 | 0.4 (-1.7, 2.6)  n=503 | 0.677 | YES | |
| Change (b-4m) | -1.3 n=246 | -0.6 n=285 |  |  |  | |
| Change (b-8m) | -1.4 n=247 | -1.4 n=272 |  |  |  | |
| **EPIC-26 (32) (higher=better)** | | | | | |  |
| Urinary incontinence (0 to 100) | | | | | |  |
| Baseline | 83.3 (20.3) n=285 | 80.3 (22.3) n=324 |  |  |  | |
| 4 months | 84.4 (19.7) n=259 | 81.8 (19.6) n=288 | 0.6 (-1.6, 2.9)  n=510 | 0.57 | YES | |
| 8 months | 84.9 (19.6) n=253 | 82.0 (19.8) n=281 | 0.4 (-1.9, 2.9)  n=500 | 0.694 | YES | |
| Change (b-4m) | -0.8 n=245 | -0.8 n=279 |  |  |  | |
| Change (b-8m) | -1.2 n=242 | -1.5 n=273 |  |  |  | |
| Urinary irritative/obstructive (0 to 100) | | | | | |  |
| Baseline | 87.3 (13.2) n=282 | 85.9 (14.3) n=318 |  |  |  | |
| 4 months | 87.8 (13.7) n=257 | 87.5 (14.8) n=275 | -1.0 (-3.2, 1.2)  n=490 | 0.364 | NO | |
| 8 months | 88.2 (13.2) n=249 | 87.7 (14.8) n=267 | -0.5 (-2.7, 1.6)  n=480 | 0.622 | NO | |
| Change (b-4m) | 0.1 n=240 | -1.2 n=264 |  |  |  | |
| Change (b-8m) | -0.5 n=239 | -1.3 n=255 |  |  |  | |
| Bowel (0 to 100) | | | | | |  |
| Baseline | 88.5 (17.3) n=290 | 87.5 (17.0) n=324 |  |  |  | |
| 4 months | 90.4 (14.6) n=262 | 86.7 (18.3) n=286 | 2.7 (0.5, 4.9)  n=490 | **0.016** | YES | |
| 8 months | 90.2 (14.8) n=254 | 87.7 (14.8) n=267 | 3.6(1.2, 6.1)  n=506 | **0.003** | YES | |
| Change (b-4m) | -1.5 n=250 | 0.8 n=279 |  |  |  | |
| Change (b-8m) | -1.2 n=247 | 0.7 n=275 |  |  |  | |
| Sexual (0 to 100) | | | | | |  |
| Baseline | 20.7 (21.6) n=268 | 18.442 (20.998) n=305 |  |  |  | |
| 4 months | 21.8 (21.2) n=242 | 19.4 (21.4) n=277 | 0.1 (-2.0, 2.4)  n=474 | 0.871 | YES | |
| 8 months | 22.3 (21.5) n=236 | 19.6 (21.2) n=264 | 0.6 (-1.9, 3.2)  n=463 | 0.618 | YES | |
| Change (b-4m) | -1.5 n=226 | -1.2 n=264 |  |  |  | |
| Change (b-8m) | -2.1 n=223 | -1.2 n=254 |  |  |  | |
| Hormonal (0 to 100) | | | | | |  |
| Baseline | 77.7 (19.8) n=283 | 78.0 (21.3) n=324 |  |  |  | |
| 4 months | 80.6 (17.5) n=258 | 80.1 (20.5) n=288 | 0.6 (-1.4, 2.7)  n=509 | 0.557 | YES | |
| 8 months | 81.4 (19.3) n=255 | 81.5 (19.9) n=276 | -0.7 (-3.0, 1.4)  n=497 | 0.491 | NO | |
| Change (b-4m) | -2.3 n=243 | -0.5 n=282 |  |  |  | |
| Change (b-8m) | -2.8 n=243 | -1.8 n=270 |  |  |  | |
| **FACT-G (33) (higher= better)** | | | | | |  |
| Total score (0 to 108) | | | | | |  |
| Baseline | 89.4 (13.4) n=279 | 87.6 (15.2) n=315 |  |  |  | |
| 4 months | 89.2 (13.1) n=254 | 87.4 (14.) n=289 | -0.04 (-1.6, 1.5)  n=499 | 0.96 | NO | |
| 8 months | 88.4 (14.2) n=258 | 87.2 (14.6) n=283 | -0.15 (-1.7, 1.4)  n=497 | 0.849 | NO | |
| Change (b-4m) | 1.1 n=237 | 1.0 n=278 |  |  |  | |
| Change (b-8m) | 1.2 n=243 | 0.8 n=270 |  |  |  | |
| Physical well-being (0 to 28) | | | | | |  |
| Baseline | 24.5 (4.0) n=287 | 24.3 (4.2) n=321 |  |  |  | |
| 4 months | 24.8(3.6) n=265 | 24.15 (4.2) n=297 | 0.4 (-0.02, 0.8) n=523 | 0.064 | YES | |
| 8 months | 24.5 (4.2) n=261 | 24.0 (4.3) n=285 | 0.2 (-0.2, 0.7) n=510 | 0.391 | YES | |
| Change (b-4m) | -0.00 n=250 | 0.4 n=289 |  |  |  | |
| Change (b-8m) | 0.24 n=250 | 0.4 n=276 |  |  |  | |
| Social/Family well-being (0 to 28) | | | | | |  |
| Baseline | 21.7 (4.5) n=288 | 21.3 (4.9) n=327 |  |  |  | |
| 4 months | 21.0 (4.9) n=265 | 21.0 (5.7) n=298 | 0.004 (-0.7, 0.7)  n=531 | 0.951 | YES | |
| 8 months | 21.0 (4.9) n=260 | 21.2 (5.2) n=290 | -0.01 (-0.7, 0.6) n=521 | 0.966 | NO | |
| Change (b-4m) | 0.6 n=253 | 0.4 n=294 |  |  |  | |
| Change (b-8m) | 0.5 n=252 | 0.2 n=285 |  |  |  | |
| Emotional well-being (0 to 24) | | | | | |  |
| Baseline | 20.5 (3.2) n=289 | 20.2 (3.7) n=323 |  |  |  | |
| 4 months | 20.5 (3.4) n=262 | 20.5 (3.5) n=297 | -0.2 (-0.7, 0.1)  n=523 | 0.219 | NO | |
| 8 months | 20.5 (3.5) n=259 | 20.2 (3.7) n=290 | 0.01 (-0.4, 0.5)  n=517 | 0.953 | YES | |
| Change (b-4m) | 0.2 n=250 | -0.1 n=289 |  |  |  | |
| Change (b-8m) | 0.07 n=251 | 0.06 n=282 |  |  |  | |
| Functional well-being (0 to 28) | | | | | |  |
| Baseline | 22.5 (5.2) n=293 | 21.8 (6.1) n=329 |  |  |  | |
| 4 months | 22.6 (5.0) n=266 | 21.6 (6.1) n=299 | -0.06 (-0.7, 0.6) n=536 | 0.85 | NO | |
| 8 months | 22.4 (5.3) n=261 | 21.7 (5.7) n=293 | 0.06 (-0.5, 0.7) n=528 | 0.848 | YES | |
| Change (b-4m) | 0.3 n=257 | 0.3 n=295 |  |  |  | |
| Change (b-8m) | 0.3 n=255 | 0.1 n=289 |  |  |  | |
| **GHQ12 (34) (higher= worse)** | | | | | |  |
| Total score (0 1 1 1 method) (0 to 12) | | | | | |  |
| Baseline | 1.1 (2.1) n=292 | 1.3 (2.4) n=333 |  |  |  | |
| 4 months | 0.9 (1.9) n=267 | 1.3 (2.4) n=301 | -0.3 (-0.6, -0.03)  n=539 | **0.032** | YES | |
| 8 months | 0.9 (2.0) n=262 | 1.1 (2.3) n=289 | -0.08 (-0.3, 0.2) n=526 | 0.583 | YES | |
| Change (b-4m) | 0.1 n=257 | -0.1 n=299 |  |  |  | |
| Change (b-8m) | 0.1 n=256 | 0.1 n=287 |  |  |  | |
| Total score (0 1 2 3 method) (0 to 36) | | | | | |  |
| Baseline | 10.0 (4.4) n=292 | 10.0 (4.7) n=333 |  |  |  | |
| 4 months | 9.5 (4.0) n=267 | 10.1 (4.5) n=301 | -0.5 (-1.1, 0.01)  n=539 | 0.057 | YES | |
| 8 months | 9.7 (4.3) n=262 | 9.8 (4.2) n=289 | -0.3 (-0.9, 0.2)  n=526 | 0.246 | YES | |
| Change (b-4m) | 0.1 n=257 | -0.3 n=299 |  |  |  | |
| Change (b-8m) | 0.3 n=256 | 0.03 n=287 |  |  |  | |
| **Worry of cancer (35) (higher=worse) (0 to 20)** | | | | | |  |
| Baseline | 6.9 (4.4) n=288 | 7.2 (5.0) n=333 |  |  |  | |
| 4 months | 6.3 (4.5) n=265 | 6.1 (4.8) n=300 | 0.36 (-0.2, 0.9)  n=533 | 0.224 | NO | |
| 8 months | 6.0 (4.4) n=261 | 6.1 (4.7) n=286 | 1.2 (-0.8, 3.4)  n=514 | 0.246 | NO | |
| Change (b-4m) | 0.4 n=253 | 0.9 n=298 |  |  |  | |
| Change (b-8m) | 0.9 n=251 | 1.0 n=283 |  |  |  | |
| **Lifestyle (higher=better)** | | | | | |  |
| Number of fruits + vegetables (38) (0 to 10) | | | | | |  |
| Baseline | 5.3 (1.9) n=286 | 4.9 (2.0) n=329 |  |  |  | |
| 4 months | 5.2 (1.9) n=266 | 4.9 (2.1) n=301 | -0.04 (-0.3, 0.2)  n=532 | 0.752 | NO | |
| 8 months | 5.3 (2.0) n=260 | 4.7 (2.0) n=289 | 0.6 (-1.6, 2.9)  n=510 | 0.57 | YES | |
| Change (b-4m) | 0.08 n=252 | -0.02 n=296 |  |  |  | |
| Change (b-8m) | 0.01 n=250 | 0.2 n=286 |  |  |  | |
| Exercise (39) (0 to 400) | | | | | |  |
| Baseline | 28.0 (23.5) n=270 | 24.6 (21.6) n=316 |  |  |  | |
| 4 months | 29.7 (27.3) n=263 | 25.3 (28.8) n=283 | 0.6 (-4.1, 5.4) n=489 | 0.783 | YES | |
| 8 months | 28.5 (27.1) n=257 | 24.7 (23.7) n=282 | -1.0 (-3.2, 1.2)  n=490 | 0.364 | NO | |
| Change (b-4m) | -1.3 n=234 | -0.9 n=271 |  |  |  | |
| Change (b-8m) | -0.4 n=232 | -0.2 n=266 |  |  |  | |
